# Optical whispering-gallery mode barcodes for high-precision and wide-range temperature measurements

**DOI:** 10.1038/s41377-021-00472-2

**Published:** 2021-02-05

**Authors:** Jie Liao, Lan Yang

**Affiliations:** 1 Department of Electrical & Systems Engineering, Washington University in St. Louis, MO 63130 St. Louis, USA; 2 Department of Physics, Washington University in St. Louis, MO 63130 St. Louis, USA

**Keywords:** Imaging and sensing, Optical sensors

## Abstract

Temperature is one of the most fundamental physical properties to characterize various physical, chemical, and biological processes. Even a slight change in temperature could have an impact on the status or dynamics of a system. Thus, there is a great need for high-precision and large-dynamic-range temperature measurements. Conventional temperature sensors encounter difficulties in high-precision thermal sensing on the submicron scale. Recently, optical whispering-gallery mode (WGM) sensors have shown promise for many sensing applications, such as thermal sensing, magnetic detection, and biosensing. However, despite their superior sensitivity, the conventional sensing method for WGM resonators relies on tracking the changes in a single mode, which limits the dynamic range constrained by the laser source that has to be fine-tuned in a timely manner to follow the selected mode during the measurement. Moreover, we cannot derive the actual temperature from the spectrum directly but rather derive a relative temperature change. Here, we demonstrate an optical WGM barcode technique involving simultaneous monitoring of the patterns of multiple modes that can provide a direct temperature readout from the spectrum. The measurement relies on the patterns of multiple modes in the WGM spectrum instead of the changes of a particular mode. It can provide us with more information than the single-mode spectrum, such as the precise measurement of actual temperatures. Leveraging the high sensitivity of WGMs and eliminating the need to monitor particular modes, this work lays the foundation for developing a high-performance temperature sensor with not only superior sensitivity but also a broad dynamic range.

## Temperature measurement: optical barcodes from whispering-gallery sensors

Extremely precise measurement of temperature using devices known as whispering-gallery mode (WGM) sensors can be greatly improved using a technique that simultaneously monitors different modes, or patterns, of the optical signals. WGM sensors rely on the sustained circulation of light within closed concave microstructures—discs, rings, or spheres—similar to the movement of sound waves in whispering galleries such as the dome of St. Paul’s Cathedral in London. Jie Liao and Lan Yang at Washington University in St. Louis, Missouri, USA, developed procedures to analyse the effect of temperature on the collective patterns of light signals in WGM sensors. The results are converted into optical barcodes which indicate the temperature directly. This multimode system overcomes significant limitations imposed by the restricted range and less direct monitoring methods of existing single-mode sensors.

## Introduction

As a fundamental physical parameter, temperature plays an important role in many physical, chemical, and biological systems. Precise thermal sensing has thus become of great interest in many scientific, engineering, and industrial areas, such as novel materials^1–3^, energy harvesting^4,5^, biomedical studies and healthcare treatment^6–11^, and environmental monitoring^12–14^. For instance, precise and continuous monitoring of temperature changes in the human body is critical in understanding the thermal phenomenon of homeostasis and providing essential diagnostic information to identify appropriate treatment protocols for diseases such as COVID-19^15^, traumatic brain injury^16,17^, and cancer^18–20^. Another example is temperature sensors on artificial intelligence robots, which help robots detect the environmental temperature. Through synchronous motility and sensory perception, robots can adapt to changing environments by actively responding to temperature and other stimuli^21–24^.

To meet these increasing demands, numerous technologies and devices for thermal sensing have been developed; however, most of them are not capable of measuring the temperature with high sensitivity and a large-dynamic range in different environments. Conventional thermometers are not able to measure small temperature fluctuations^25,26^, especially on the submicron scale, such as in microcircuits and intracellular liquids. Among various conventional thermal-sensing systems, optical sensing technologies are finding increasing interest and applications due to their selectivity, immunity to electromagnetic interference and the capability for multiplexing and remote sensing. For the past few decades, various optical structures, such as waveguides^27–29^, photonic crystal fibres^30–32^, and Sagnac interferometers^33–35^, have been developed for thermal sensing. However, accurate temperature sensing with high resolution based on single-pass (waveguide or fibre) optical sensors is challenging due to the limited optical sensing path. In those sensors, light interacts with the sensing material only once; consequently, thermal sensitivity and response are generally limited by the dimension of the sensor.

An optical resonator^36^ is a promising candidate as a solution to overcome the aforementioned limitations. Light could circulate in a high-quality resonator over millions of times, thereby significantly enhancing the light-matter interactions and consequently improving the sensitivity^37–40^. In addition, the high-quality factor (*Q*-factor) of a resonator leads to a narrow bandwidth in the spectrum, making it easier to resolve subtle changes when tracking the resonant wavelengths, which consequently improves the detection limit^37,41^. Among various kinds of optical resonator sensors, whispering-gallery mode (WGM) resonators have attracted increasing attention due to their exceptionally high-quality factors^42–44^. Various WGM resonators have been demonstrated to measure the temperature in both laboratory and outdoor environments by tracking the changes in the resonant wavelength^45–49^ because the temperature dependence of the resonance arises from thermally induced changes in the refractive index and in the physical dimensions of the resonator. This sensing method has been used for different thermal sensing applications^46,47,50,51^.

However, despite its potential sensitivity and resolution, there are certain limitations in tracking the resonance of particular modes in sensing experiments. First, the actual temperature cannot be extracted from the WGM spectrum directly. In previous approaches, sensing is achieved by monitoring the relative shift of a resonant mode induced by a temperature change with respect to its original state (baseline); therefore, it is impossible to determine the absolute value of the temperature only from the WGM spectrum without knowing the initial temperature. Second, the dynamic range of the measurement is limited by the requirement to continuously scan the laser around the same mode that changes with temperature. The previous methods can no longer track the resonance if the mode shifts out of the scanning range. Although the dynamic range can be improved by increasing the laser scanning range, the resolution will drop accordingly since the number of data points for each spectrum is limited. Consequently, there is an urgent need for optical sensor technologies that enable direct temperature measurement with both a large-dynamic range and high resolution in a reliable way.

To address these challenges, we propose a new approach to obtain information from the collective behaviour of multiple modes in the WGM spectrum. The transmission spectrum of a WGM resonator has distinct spectral features (resonant wavelengths, mode spacing, coupling depths, linewidths, etc.) at different temperatures. In other words, the temperature uniquely determines the overall pattern of the spectrum. Therefore, we could derive the actual temperature from the overall pattern of the spectrum. It is worth noting that this measurement is not limited to specific groups of modes. Based on the WGM spectrum patterns, we develop a barcode-based sensing technique that involves collective multimode information to measure the temperature directly from the WGM spectrum. This will overcome the limitations of conventional thermal sensing based on single-mode tracking.

## Results

### The optical WGM barcode and direct temperature readout

**Fig. 1:**
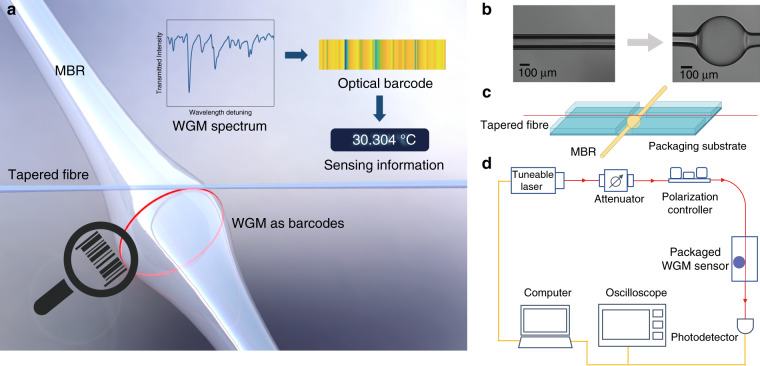
Optical WGM barcode sensing system. **a** Schematic of WGM sensing using the optical barcode technique. The light is coupled into and out of the MBR via a tapered fibre. We can directly obtain the sensing information from the optical barcode generated from the transmission spectrum. **b** Optical micrograph of the capillary (left) and fabricated MBR (right). The MBR is in the middle supported by capillaries on both sides. **c** Schematic of the packaged substrate containing the MBR and the tapered fibre. **d** Schematic of the setup. The light from a tuneable laser is used to probe the spectrum of the packaged WGM sensor. The light intensity is controlled by the attenuator and the polarization is controlled by the polarization controller. The transmission spectrum is received by a photodetector, which is connected to an oscilloscope and a computer with a data acquisition card for control of laser scanning and signal processing

The WGM barcode sensing mechanism relies on analysing the collective pattern of the WGM spectrum, which is determined by the temperature (Fig. 1a). Before an actual temperature measurement, multiple spectra are recorded and characterized as standard barcodes at different temperatures for calibration. Then, at a particular temperature, the spectrum of the sensor is measured, and an optical barcode is subsequently generated. By comparing its collective pattern with the barcode patterns in the pre-calibrated database and searching for the pattern with the best overlap, the actual temperature can be determined. To achieve this, we use the cross-correlation function to quantitatively estimate the similarity and relative collective shift of two barcodes. Suppose the measured barcode vector is *x*_*n*_ and that of the standard barcode in the database to be compared is *y*_*n*_. The cross-correlation function calculates the collective shift between them and can be used to evaluate their similarities:

1 $$R_{xy}(m) = \left\{ {\begin{array}{*{20}{c}} {\mathop {\sum}\limits_{n = 0}^{N - m - 1} {x_{n + m}y_n^ \ast ,} } & {m \ge 0} \\\!\!\!\!\!\!\!\!\!\!\!\!\! {R_{yx}^ \ast ( - m),} & {m \;<\; 0} \end{array}} \right.$$

where *m* is the shift index and *N* is the number of elements in the barcode array. If *x*_*n*_ and *y*_*n*_ are similar, then the largest element in *R* is located at the shift value where the elements of x and y best match; otherwise, *R* is a null matrix. Using the cross-correlation function, we can find the best overlapping barcode and derive the actual temperature by taking into account the small temperature deviation (relative collective shift). This is a direct measurement of the actual temperature rather than a measurement of the relative temperature difference. Additionally, since we do not rely on tracking the shift of particular modes, this sensing would still be applicable even if particular modes shift out of the spectrum in the measurement. Microbubble resonators (MBRs) are suitable for our proposed sensing mechanism since they typically have a dense WGM spectrum resulting from their highly oblate geometry, which supports many non-degenerate WGMs^52,53^. The MBR shown in Fig. 1b is packaged on a substrate (Fig. 1c) to improve the robustness and stability of the entire sensor system^54^. The WGM spectrum is measured using the apparatuses illustrated in Fig. 1d.

**Fig. 2:**
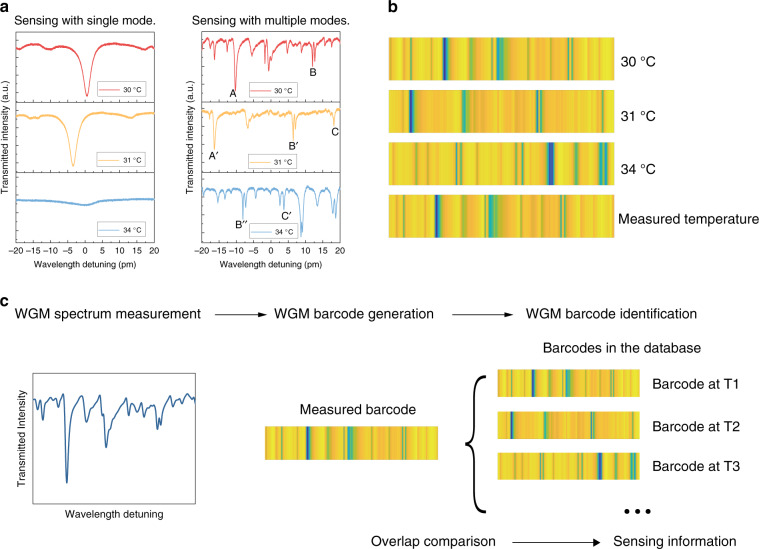
Demonstration of the WGM barcode technique. **a** A comparison between the WGM spectra for the conventional single-mode tracking method (left) and the proposed barcode sensing with multiple modes (right). In the WGM spectra for single-mode tracking, tracking of resonance is not possible as the mode shifts out of the scanning range at 34 °C. In the WGM spectra for barcode sensing with multiple modes, the resonant wavelengths, spacing between modes, linewidths, and coupling depths are determined by the temperature. **b** Optical barcodes generated at different temperatures. Each line represents a mode from the spectrum. The resonant wavelengths, linewidths, coupling depths, and spacing between modes are converted to the positions, widths, colours, and distance between lines in the barcode. The measured temperature should be close to 30 °C or 31 °C, rather than 34 °C, based on their pattern similarities. **c** Workflow of sensing with optical barcodes. During measurement, the WGM spectrum is measured, and a barcode is subsequently generated. By comparing the pattern of the measured barcode with the barcodes in the database, the sensing information can be extracted directly

A comparison between the single-mode tracking and the proposed barcode sensing with multiple modes is shown in Fig. 2a, b. The single-mode spectrum is from a microtoroid resonator with a sparse spectrum^55^, while the multimode spectrum is from the MBR. The spectra of both resonators are measured at 30 °C, 31 °C, and 34 °C. For single-mode tracking, the mode is only identifiable and trackable within a small temperature change (30–31 °C). It is impossible to further track the shift as the mode moves out of the scanning range. For our proposed sensing mechanism involving the collective multimode pattern, on the other hand, we do not rely on tracking the shift of particular modes. Instead, we generate barcodes from a multimode spectrum that are uniquely associated with temperature to obtain sensing information by data analysis. In this new approach, in addition to the changes in resonant wavelengths, the variations in other modal characteristics, such as the spacing between modes, linewidths, and coupling depths, are all included in our analysis. For instance, mode A at 30 °C evolves into mode A’ when the temperature rises to 31 °C. Besides resonance shift, its linewidth and coupling depth also change. The linewidth decreases from 0.6252 pm to 0.5164 pm, and the resonance depth changes from 0.148 to 0.392. At 34 °C, this mode shifts out of the scanning range. Similarly, mode B evolves into mode B′ and further changes to mode B″. In addition, at 31 °C, a new mode (mode C) emerges and evolves into mode C′ at 34 °C. These changes in linewidth and coupling strength (depth) are caused by the variation in the coupling gap distance between the fibre and the MBR, induced by the thermal expansion of the material. The variations in spacing are due to the different thermal responses of different modes. As a result, these multiparameter changes of modes can be regarded as a collective behaviour, which ultimately changes the overall pattern of the spectrum. To make full use of the extra information provided by the multimode spectrum, the optical WGM barcode should reflect not only the mode positions but also the linewidth and the coupling depth in the spectrum. To achieve this, the transmission intensity in the spectrum is divided into 10,000 pieces to form a one-dimensional array. Each element of the array corresponds to a rectangular area in the barcode image, whose colour is determined by the value of the element through a colormap. The colormap used here is the Parula colormap, which can maintain a smooth colour gradient even when plotted in greyscale. The WGM barcodes at various temperatures are generated, as shown in Fig. 2b. The optical barcode consists of multiple lines, and each line represents a mode from the spectrum. The width of each line indicates the linewidth of the mode, and the colour reflects its coupling depth. Apparently, the barcode patterns at different temperatures are unique and distinctive, which supports our hypothesis that the collective pattern of the multimode spectrum is uniquely determined by the temperature.

Using the WGM barcode technique, the actual temperature can be directly obtained from the multimode spectrum. We demonstrate the sensing process by measuring the spectrum at a particular temperature. As shown in Fig. 2c, a barcode is generated from the measured spectrum. By comparing its pattern with the standard patterns in the database, we know that it best matches the barcode at 30 °C with a small deviation (shift) of 1.367 pm. Using the sensitivity of 4.5 pm/°C, the actual temperature should be approximately 30.304 °C, which is further confirmed by the readout of the surface resistance temperature detector of 30.3 °C. In this way, we can directly measure the actual temperature, and the measurement is not limited to the scanning range of the laser. The generated barcode also provides an instinctive method for data visualization. It is easy to tell that the measured temperature in Fig. 2c should be close to 30 °C or 31 °C, rather than 34 °C, based on their pattern similarities.

To ensure that we can obtain a WGM spectrum with rich spectral features across the free spectral range (FSR) of the MBR, we measure the spectrum of the MBR (FSR ~ 0.14 nm) over a wavelength range of 1 nm, and the number of modes is 305 (see Fig. S1 (a) in the supplementary information). Based on this measurement, the average spacing between two modes is approximately 3.3 pm, and the average number of modes within the laser scanning range (40.56 pm) is 12. Therefore, we can always observe multiple modes in the WGM spectrum. This high modal density of the MBR makes it a suitable platform for sensing based on the multimode spectrum.

### Reliability and sensitivity

**Fig. 3:**
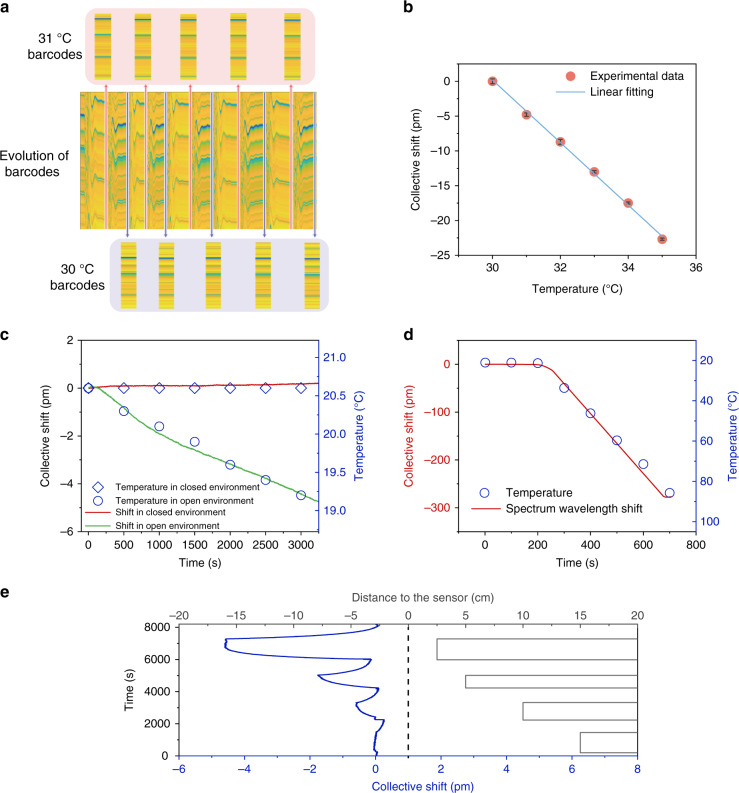
Characterization of the sensing system based on WGM barcodes. **a** Evolution of optical barcodes between 30 °C and 31 °C for 5 cycles. **b** Linear dependence of the collective WGM shift on the temperature, where the red balls denote experimental measurements and the blue curve is a linear fit to the experimental results. **c** Detection of temperature drift. The red curve denotes the small collective shift induced by temperature fluctuations when the sensor is placed in a thermally isolated environment. The blue squares are the temperatures measured by a thermometer. The green curve denotes the relatively large shift when the sensor is placed in an open environment, and the blue circles are the measured temperatures. **d** Large-dynamic range of the measurement. The red curve denotes the collective shift in response to a large temperature change. The blue circles are the recorded temperature. **e** Temperature sensing of a warm object (45 °C) at different distances. Blue curve: collective shift as the object is placed at various distances. Grey line: position of the object with respect to the sensor surface marked by the dashed line at 0

The optical WGM barcode technique relies on one-to-one mapping between the pattern of the spectrum and the temperature, so the repeatability of patterns at the same temperature is critical. We confirm this hypothesis by switching the local temperature between 30 °C and 31 °C for multiple cycles and comparing the patterns of WGM barcodes at the same temperature. In each cycle, as the temperature is switched, the proportional–integral–derivative (PID) controller compares the current temperature and the temperature set point and calculates the desired actuator output for the heater. This process is repeated continuously, and the temperature gradually reaches the set point^56^. As shown in Fig. 3a, in general, there is little difference between the barcodes obtained at the same temperatures. Although some of the barcodes at 31 °C seem blurry (increased linewidth), the algorithm of the cross-correlation between them shows a standard deviation of 0.229 pm and a maximum difference of 0.595 pm (corresponding to a temperature difference of ~0.132 °C), which is around the level of the temperature stability of the PID thermal control (~0.1 °C). This may come from the small temperature deviation beyond the accuracy of the thermal control. Based on this information, we can estimate the measured temperature in Fig. 2c as 30.304 ± 0.132 °C.

To determine the sensitivity of our sensor, the local temperature is kept at different values (30–35 °C in 5 steps) for 150 s. The collective shift is derived from the cross-correlation function. As shown in Fig. 3b, a total shift of 22.677 pm induced by the 5.0 °C temperature change is observed. The calculated sensitivity obtained from linear fitting is 4.5 pm/°C. It is worth noting that for packaged WGM thermal sensors, the sensitivity will be modified by the packaging material. In our case, the overall spectrum experiences a blueshift with increasing temperature due to the opposite signs of the thermo-optical coefficients of UV glue (negative) and silica materials (positive). In addition, for the same sensor, different modes exhibit various sensitivities due to the different modal distributions. Therefore, in conventional WGM thermal sensors, the strategy is to find and track the mode with high sensitivity, which requires laborious work during calibration, and we may not be able to locate the same mode every time. In contrast, in the WGM barcode measurement that measures the overall shift of a multimode spectrum, the sensitivity does not rely on the sensitivity of specific modes; instead, it depends on the average shift of all the modal features. The collective blueshift of the spectrum implies that most of the WGMs are affected by the UV glue with a negative thermo-optical coefficient.

### High resolution and large-dynamic range

The high *Q*-factor of the WGM sensor contributes to the high resolution in sensing. To show the capability of resolving small temperature changes, we measure the collective shift when the sensor is placed in a thermally isolated environment and an open environment. A thermometer with a detection resolution of 0.1 °C is mounted along with the WGM sensor for comparison. As shown in Fig. 3c, in an open environment, the temperature decreases by approximately 1.4 °C, and the measured collective shift well matches the result from the commercial thermometer, while in a temperature-maintaining environment, the WGM barcode sensor can still detect temperature fluctuations as small as 0.04 °C over 3000 s, which is beyond the detection limit of the thermometer. The linewidth of the high-*Q* mode used here is approximately 0.070 pm (see Fig. S1b in the supplementary information). Assuming that the smallest resolvable change in the resonance is 1/10 of the linewidth, the detection limit can reach as low as 0.002 °C. With better resolution and the capability for real-time monitoring, the optical barcode sensor shows great advantages in detecting extremely small temperature changes.

To explore the feature of large-dynamic range, we place the sensor in water on a hot plate. The water is heated, and blueshifts are observed in the WGM spectrum with increasing temperature. The WGM collective shift, as well as the temperature change, is shown in Fig. 3d. The temperature difference is large (~65 °C), and the overall WGM spectrum shift (~275 pm) is much larger than the laser scanning range of ~40.56 pm, which is determined by the maximum modulation voltage we apply to the laser. The results prove that the WGM barcode sensing technique provides an effective way to track the collective shift of the spectrum. With this method, the dynamic range is no longer limited by the scanning range of the laser, which will significantly enhance the dynamic range of the measurement. Ultimately, the dynamic range may be limited by the materials of the structure, depending on the temperature change the sensor can sustain. As long as the sensor does not suffer from irreversible damage and provides multimode spectra, we can implement the barcode technique for sensing.

To further explore the sensing performance of our sensor, we characterize the response of our sensor when exposed to a heating source at different distances. A small heating element with a size of 18.0 mm × 18.0 mm is heated to ~45 °C. This temperature is determined and maintained constant by a thermometer during the measurements. The heating element is placed 15 cm from the sensor for ~1000 s and then is moved away. This test is also repeated at distances of 10, 5 and 2.5 cm. As shown in Fig. 3e, the results demonstrate that the sensor is able to detect small warm objects (approximately the size of a finger or a small insect) at a distance of 15 cm.

### Real-time monitoring of the evaporation of droplets

**Fig. 4:**
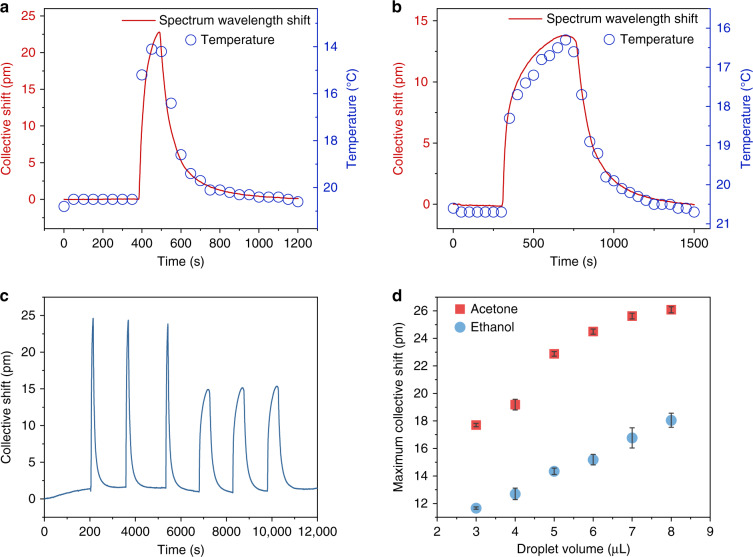
Monitoring the spontaneous evaporation of liquid droplets. **a** Transient dynamics as an acetone droplet of 5 µL is positioned on the sensing surface. Red curve: collective shift of the spectrum. Blue circles: measured temperature. **b** Transient dynamics as an ethanol droplet of 5 µL is positioned on the sensing surface. Red curve: collective shift of the spectrum. Blue circles: measured temperature. **c** Sensorgram of droplet evaporation. The first three peaks are the response for 5 µL acetone droplets, and the last three peaks are the response for 5 µL ethanol droplets. **d** Maximum collective shifts in response to the evaporation of acetone and ethanol droplets of various volumes (3–8 µL in 5 steps repeated 3 times). With a larger volume, the droplet would consume more heat from the surroundings and lead to a larger temperature change (maximum shift). For the same droplet volume, acetone causes a larger temperature change than ethanol during evaporation

With the small footprint, high resolution, large-dynamic range, and the ability for real-time measurement, the WGM barcode sensor is a powerful platform to study transient thermal dynamics. To demonstrate this, we use our sensor to measure the temperature dynamics induced by spontaneous evaporation of small liquid droplets on a substrate. We first place an acetone droplet of 5 µL on the sensing surface (surface of the packaged sensor; the MBR is approximately 200 µm below the surface) with a pipette and record the transmission spectra throughout the evaporation process. The change in temperature is also measured during the evaporation process. Fig. 4a, b shows the dynamics of the collective shifts as well as the temperature changes during the spontaneous evaporation of acetone and ethanol droplets (5 µL). The temperature of the substrate drops immediately when the droplet touches the surface, leading to a redshift of the WGM spectrum. The temperature drop rate decreases as the droplet evaporates. Finally, the temperature returns to the initial level (as does the spectrum). These transient thermal dynamics are related to the intrinsic thermal properties of the liquid (such as the evaporation rate, thermal conductivity, and molar specific heat capacity), so the two sensorgrams show distinctive dynamics of the temperature during evaporation^57–59^. Acetone has a larger evaporation rate than ethanol, so the thermal response changes faster than that with ethanol^57^. To ensure the reliability, we repeat the measurement of spontaneous evaporation of droplets. As shown in Fig. 4c, the first three peaks in the sensorgram are the response for acetone droplets, and the last three peaks are the response for ethanol droplets. Apparently, there is little difference between the response patterns for the same droplet composition. They exhibit similar response curves, similar maximum shifts, and similar durations. Although the mass of droplet could also cause changes in the spectrum, our control experiment shows that the collective shift induced by mass change is negligible (see Fig. S1c in the supplementary information).

The maximum collective shift reflects the maximum temperature change during the evaporation. We measure droplets of different volumes (3–8 µL in 5 steps) and repeat the experiments three times for each volume. The maximum collective shifts are shown in Fig. 4d. It is obvious that with a larger volume, the droplet would consume more heat from the surroundings and lead to a larger temperature change. Additionally, for the same droplet volume, acetone would cause a larger temperature change than ethanol during evaporation. Based on their distinctive behaviours in the evaporation-induced transient temperature fluctuations, we could obtain more information on the dynamics in droplet evaporation. The WGM barcode sensor shows potential as a high-performance sensing platform to study the evaporation and other transient thermal dynamics of liquids or microstructures^60–64^.

## Discussion

In summary, we have demonstrated an optical WGM barcode technique for direct readout of the actual temperature from the multimode spectrum with high precision. The sensitivity of the WGM sensor based on multimode sensing is 4.5 pm/°C, and the detection limit can reach as low as 0.002 °C. We further demonstrate the measurement of a large temperature change and observe a large collective shift of over 275 pm, far exceeding the fine scanning limitation of the laser in our experiments. This result shows that the WGM barcode technique is capable of analysing the spectrum over large ranges and overcoming the limitation in the dynamic range of previous WGM sensors without sacrificing the resolution. In addition, a non-contact temperature measurement is also demonstrated, which makes the technique highly useful in hazardous or corrosive environments. Finally, we show how real-time temperature measurement using our sensor enables monitoring of spontaneous evaporation of microdroplets. The results reveal opportunities in the study of transient thermal dynamics of physical and chemical processes. It is worth noting that our optical WGM sensor is not limited to thermal sensing; it can also be implemented in biochemical sensing^65–69^, nanoparticle detection^70–72^, magnetic detection^73–75^, photoacoustic detection^76,77^, and many other applications with a proper design. For a resonator-based magnetic field sensor, for instance, the resonance, coupling depth, and linewidth change with the magnetic field intensity^74,75^ and therefore can be used as barcode features. Using the barcode technique, we can measure a higher magnetic field intensity without changing any hardware. Even if some modes shift out of the scanning range, we can still determine the magnetic field intensity based on the pattern of the spectrum.

The WGM barcode technique integrates high-sensitivity, high-resolution, and large-dynamic-range measurement into a high-performance sensing platform without the need for extra complicated designs and expensive components. In addition, the technique can, in principle, be adopted for other types of optical resonant-based sensors with rich modal features, such as surface plasmon resonance sensors^67,78^, photonic crystal sensors^75,79^, inline fibre interferometers^34^, and even fluorescent and lasing-based sensors^68,80,81^. Therefore, our simple and general technique could have a variety of promising applications, ranging from physical thermodynamics to chemical thermodynamics, robotic sensing, and thermal phenomena in biomedical research. Some limitations arise from the need for polarization control to ensure the same spectrum. The implementation of polarization-maintaining optical fibres^82^ is a common practice and can offer a direct solution to this problem. To further enhance the sensitivity, materials with a large thermo-optical coefficient^50,83^ can be injected into the core of the MBRs. Moreover, noise-suppression techniques, such as power and frequency stabilization of lasers^84,85^ and self-referencing techniques^86,87^, can be implemented to further improve the detection limit.

## Materials and methods

### Device fabrication

The MBRs are fabricated from silica capillaries by a “heat and expand” approach^88^. Before fabrication, we burn out the polymer coating on the capillary with a flame. Then, one end of the capillary is sealed, and the other end is connected to a pressure control system. Afterwards, the capillary is heated with a Vytran glass processor (~60 W), and the pressure inside is increased (~1.5 atm) simultaneously to gradually expand the capillary into a bubble shape. The diameter of the microbubble can be controlled by the pressure and the heating power^89^. The *Q*-factors lie in the range of 10^6^−10^7^. To excite WGMs in the MBR, a tapered fibre waveguide^90^ is used to couple light into and out of the resonator.

To make a compact sensing system, we use a packaging technique^88^ to encapsulate the MBR and the tapered fibre on a 3D printed substrate with dimensions of 8 × 40 × 5 mm (width × length × height). The substrate has two channels perpendicular to each other, so the MBR and the tapered fibre can be suspended inside the channels and the light from the tapered fibre can be coupled into the MBR. Droplets of low refractive index UV curable adhesive (MY132A) are used to fill the two channels before they are cured by UV light^54,91,92^. After packaging, the sensor with a small footprint is able to maintain the coupling efficiency and *Q*-factors for a long time, and the mechanical perturbations can be greatly suppressed.

### Characterization and calibration of WGM spectra for temperature measurement

The collective pattern of the WGM spectrum is at the heart of our sensing mechanism. To obtain the spectrum, a tuneable laser source in the 780 nm band (TLB-6712) is used to scan across multiple modes, and the transmission spectrum is received by a photodetector. The 780 nm wavelength band is used to avoid large absorption in the infrared in order to obtain high *Q*-factors and consequently achieve high resolution. The signals from the photodetector are displayed on an oscilloscope for observation and connected to a computer through a data acquisition card for analysis. The data acquisition card also generates a triangle wave for wavelength scanning of the laser. The modulation voltage is ± 1 V, with a modulation frequency of 60 Hz, driving the laser scanning from −20 pm to 20 pm around the centre wavelength. This scanning range can provide high resolution to resolve subtle changes in the multimode spectrum. The light intensity is adjusted by an optical attenuator, and the polarization is controlled by a polarization controller. To achieve better stability, the laser power is kept low (~65 µW) to minimize optothermal effects, which could cause distortion and broadening of the Lorentzian lineshape^93^. The measurement time for each spectrum is ~17 ms, limited by the scanning speed of the laser modulation.

To calibrate the temperature response, the sensor is sandwiched in contact between a Peltier heater and a heatsink. A surface-mount resistance thermometer with a resolution of 0.1 °C is attached to the sensor surface to provide an additional independent measurement of the local temperature as well as PID thermal control of the heater. When the temperature under thermal control becomes stable at a set value (30 °C, 31 °C, 32 °C, etc.), the WGM spectrum is recorded for 10 s. Then, a standard barcode is generated by averaging the spectra recorded at the same temperature. Fig. 1a illustrates the concept of the WGM barcode technique. During calibration, the heater, thermometer, and sensor are placed inside a wooden cell for thermal isolation.

## Supplementary information

Supplementary information File
