# Supplementary material for: Optical whispering-gallery mode barcodes for high-precision and wide-range temperature measurements
Source: Light Sci Appl. 2021 Feb 5;10:32. doi: 10.1038/s41377-021-00472-2 (PMC7862871; doi:10.1038/s41377-021-00472-2)
Supplement: Supplementary file 1 — Supplementary information File [file 41377_2021_472_MOESM1_ESM.docx]

**Supplementary Information for “Optical whispering-gallery mode barcodes for high-precision and wide-range temperature measurements”**

Jie Liao^1^ and Lan Yang^1,2^

^1^Department of Electrical & Systems Engineering, Washington University in St. Louis, MO 63130;

^2^Department of Physics, Washington University in St. Louis, MO 63130

Correspondence: Lan Yang ([yang@seas.wustl.edu](mailto:yang@seas.wustl.edu))

**1. The spectral density of MBR spectrum**

We scan the laser wavelength over a range of 1 nm (772 nm to 773 nm), and the dense mode spectrum is shown in Fig. S1 (a). The scanning range is much larger than the FSR of the MBR (~ 0.14 nm). To avoid false alarms from noises in finding mode locations, we first smooth the spectrum with a span of 5 (the number of data points for calculating the smoothed value). Then the prominent resonant dips in the spectrum are automatically detected, using the “findpeaks” function in MATLAB^1^ with a threshold of 0.004. The resulted number of prominent modes is 305. Therefore, the average spacing between two modes is around 3.3 pm. Obviously, this average mode spacing is much smaller than the fine scanning range used in sensing (40.56 pm), which is limited by the modulation voltage applied to the laser. That means, on average, there will be 12 modes appearing in the spectrum within the fine scanning range. This high modal density of the MBR makes it a suitable platform for sensing based on the multimode spectrum.

**2. The resolution**

To estimate the resolution, we measure the linewidth of the WGM in the transmission spectrum by Lorentz fitting. As shown in Fig. S1 (b), The linewidth of the high-*Q* mode is around 0.070 pm. Suppose 1/10 of linewidth is resolvable, the detection limit of our sensor can reach as low as 0.002 ºC. The wavelength detuning between two data points in our measurement is 0.00405 pm. Ideally, the correlation calculation can resolve such a small difference. But considering that we may have larger noises in real applications, it is more practical to resolve 1/10 linewidth based on our past experiences.

**3. The droplet mass**

To estimate the collective shift induced by mass change of the droplet during evaporation, we use a cured UV glue droplet to replace the liquid droplet. The weight of this UV glue droplet is 7.3 mg, which is close to the weights of liquid droplets used in thermal sensing. (The densities of ethanol and acetone are 789 kg m^-3^ and 784 kg m^-3^. So the weights of the 8 µL ethanol and acetone droplets are 6.312 mg and 6.272 mg.) The solid UV glue is dropped on the sensor surface and then is moved away from the surface. As shown in Fig. S1 (c), the collective shift induced by the dropping of UV glue is ~ 0.015 pm, much smaller than the shift induced by the temperature change during droplet evaporation. Therefore, it is reasonable to neglect the mass change of the droplet during evaporation.

**4. Additional figures**





Figure S1. (a) WGM spectrum of the MBR with a wavelength range from 772 nm to 773 nm. The red dots indicate the prominent resonant modes in the spectrum. (b) A typical WGM spectrum of the MBR used in the measurement. The red dotted line is the Lorentz fitting to a high-*Q* mode. (c) Collective shift induced by dropping and moving away a small cured UV glue droplet.

**5. Reference**

1. Müller, A. V., Amigo, J. M., Wichmann, N. R., Witschas, F. B. & McEvoy, F. J. Fingerprinting of Doppler audio signals from the common carotid artery. *Scientific Reports* **10**, 2414 (2020).
